# Insights into the combination of neuromuscular electrical stimulation and motor imagery in a training-based approach

**DOI:** 10.1007/s00421-020-04582-4

**Published:** 2021-01-08

**Authors:** Amandine Bouguetoch, Alain Martin, Sidney Grosprêtre

**Affiliations:** 1 Cognition, Action and Sensorimotor Plasticity [CAPS], Unité INSERM 1093, Université de Bourgogne-UFR STAPS, BP 27877, 21078 Dijon Cedex, France; 2 EA-4660 C3S Culture Sport Health Society, University of Bourgogne Franche-Comté, Besançon, France

**Keywords:** Strength, Triceps surae, Ultrasonography, Spinal excitability, V-wave

## Abstract

**Introduction:**

Training stimuli that partially activate the neuromuscular system, such as motor imagery (MI) or neuromuscular electrical stimulation (NMES), have been previously shown as efficient tools to induce strength gains. Here the efficacy of MI, NMES or NMES + MI trainings has been compared.

**Methods:**

Thirty-seven participants were enrolled in a training program of ten sessions in 2 weeks targeting plantar flexor muscles, distributed in four groups: MI, NMES, NMES + MI and control. Each group underwent forty contractions in each session, NMES + MI group doing 20 contractions of each modality. Before and after, the neuromuscular function was tested through the recording of maximal voluntary contraction (MVC), but also electrophysiological and mechanical responses associated with electrical nerve stimulation. Muscle architecture was assessed by ultrasonography.

**Results:**

MVC increased by 11.3 ± 3.5% in NMES group, by 13.8 ± 5.6% in MI, while unchanged for NMES + MI and control. During MVC, a significant increase in V-wave without associated changes in superimposed H-reflex has been observed for NMES and MI, suggesting that neural adaptations occurred at supraspinal level. Rest spinal excitability was increased in the MI group while decreased in the NMES group. No change in muscle architecture (pennation angle, fascicle length) has been found in any group but muscular peak twitch and soleus maximal M-wave increased in the NMES group only.

**Conclusion:**

Finally, MI and NMES seem to be efficient stimuli to improve strength, although both exhibited different and specific neural plasticity. On its side, NMES + MI combination did not provide the expected gains, suggesting that their effects are not simply cumulative, or even are competitive.

## Introduction

It is now well established that training by resistance exercise in untrained individuals can induce muscle strength gains involving adaptations occurring within the muscle but also at the level of the neural drive (Aagaard 2003; Tallent et al. 2017; Kidgell et al. 2017). However, the significant mechanical muscle stress induced by heavy loads combined with a low number of repetitions does not seem to be the only strategy allowing strength gains in untrained subjects.

Indeed, there is now ample evidence demonstrating that motor imagery (MI), which consists in mentally simulating a movement without concomitant motor output (Jeannerod 1994), is also an effective way to improve motor performance and motor learning (Guillot and Collet 2008). This modality of training is efficient to induce force enhancements in upper and lower limb muscles (Paravlic et al. 2018). For instance, Zijdewind et al. (2003) but also Sidaway and Trzaska (2005) reported strength gains of more than 30% on the lower limbs after 4 and 7 weeks of MI training, respectively. Shorter training period with higher training frequency (daily routine) also showed significant force enhancements in the lower limb (Grosprêtre et al. 2017). However, MI solicits only a part of the neuromuscular system. Indeed, for a long time, strength gains following mental training have been attributed to a cortical reorganization only (Ranganathan et al. 2004). But more recently, it was shown that MI training may also induce plasticity at spinal level (Grosprêtre et al. 2017). The repetitive activation of cortical motor areas during MI, although at a subthreshold level since no motor output is observed, seems to impact spinal networks and more precisely the presynaptic neuronal circuitry (Grosprêtre et al. 2019). However, although a large portion of the brain-to-muscle pathway seems to be involved, MI training do not provide motor output. This demonstrates that there is room for optimizing MI-training induced gains by combining it with other training modalities (e.g., neuromuscular electrical stimulation) leading to activation/recruitment of muscle fibers.

Neuromuscular electrical stimulation (NMES) is another alternative to physical training to improve muscle strength and is a widely used method in applied sports science or rehabilitation (Maffiuletti 2010). In contrast to a typical voluntary contraction initiated by the central nervous system (e.g., in resistance training), NMES consists in evoking contractions by applying an electrical current over the muscle via surface electrodes (Seyri and Maffiuletti 2011). With submaximal levels of evoked contraction, NMES strength gains were largely demonstrated in the literature, mostly on the lower limb (Laughman et al. 1983; Gondin et al. 2005). Strength gains following NMES training (usually between 10 and 30%, Hainaut and Duchateau 1992) have been obtained without significant changes in muscle mass or architecture, especially in the early phase of the training. This indicates that nervous mechanisms are involved in this improvement (Gondin et al. 2005, 2006) as evidenced by an enhancement of the voluntary muscle activation as measured with surface electromyography (Maffiuletti et al. 2002) or twitch interpolation method (Stevens et al. 2004). These neural effects may originate from a repetitive activation of afferent nervous circuitry, from the muscle up to the central nervous system. Despite some effects on the corticospinal network being observed with NMES, the incomplete activation of the motor system during NMES and the use of submaximal training intensities (Maffiuletti 2010) should partly account for its lower efficiency on muscle strength than resistance training (Laughman et al. 1983; Hainaut and Duchateau 1992). Thus, adding an efferent command without additional training loads (i.e. MI) could improve the gains and be applicable in rehabilitation purposes.

Therefore, both MI and NMES represent an incomplete activation of the neuromuscular system, but both sufficient to induce significant neural plasticity leading to improving muscle function. Saying that, the complementary gradients of MI and NMES neural activation led to the hypothesis that alternating both treatments in the same training session (NMES + MI) may provide additional effects compared to one of these modalities alone. The repetitive activation of cortical regions and corticomotoneuronal pathway provided by MI alternated with the repetitive afferent activation and motor output provided by NMES may lead to greater neural adaptations. The combination of both could therefore represent a promising training method. This alternating treatment could lead to an improvement of maximal strength without involving high training loads associated with voluntary resistance training. However, this approach has rarely been assessed and compared to NMES or MI alone. To date, little is known in the literature whether the effects provided by both methods can be complementary, particularly in a chronic approach. Training effects of such combination are not clear, because the different mechanisms involved in NMES and MI could also be antagonist and limit strength gains when applied in the same training session. Indeed, it has been previously shown that acute effects of MI (Grosprêtre et al. 2018) and NMES (Grosprêtre et al. 2019) can induce opposite modulation of presynaptic inhibition mechanisms.

The aim of this study was to assess strength gains following a training approach combining MI and NMES, and investigating the muscular and neural plasticity that results from this combination. This was compared to NMES and MI training performed alone with the same total number of repetitions and sessions. We can hypothesize that the summation of NMES and MI effects during training sessions would lead to greater enhancement of muscle strength, with a greater neural plasticity than these treatments applied alone, despite different neural mechanisms involved.

## Methods

### Participants

Thirty-seven healthy subjects (age: 24 ± 5.8 years old; height: 174 ± 9 cm; weight: 70 ± 14 kg, 12 females) gave written informed consent to participate in the present study and complied with the whole protocol. None of them reported any neurological or muscular disorders. There was no statistical difference between the groups of age, sex and physical activity pattern. Participants were recreationally active, and were asked not to perform any intense exercise during the training period. The experimental design was approved by the regional ethic committee (CPP COOM III number 2017-A00064-49; Clinical trial.gov identifier NCT03334526) and conducted in conformity with the latest version of the Declaration of Helsinki.

### General experimental design

**Fig. 1:**
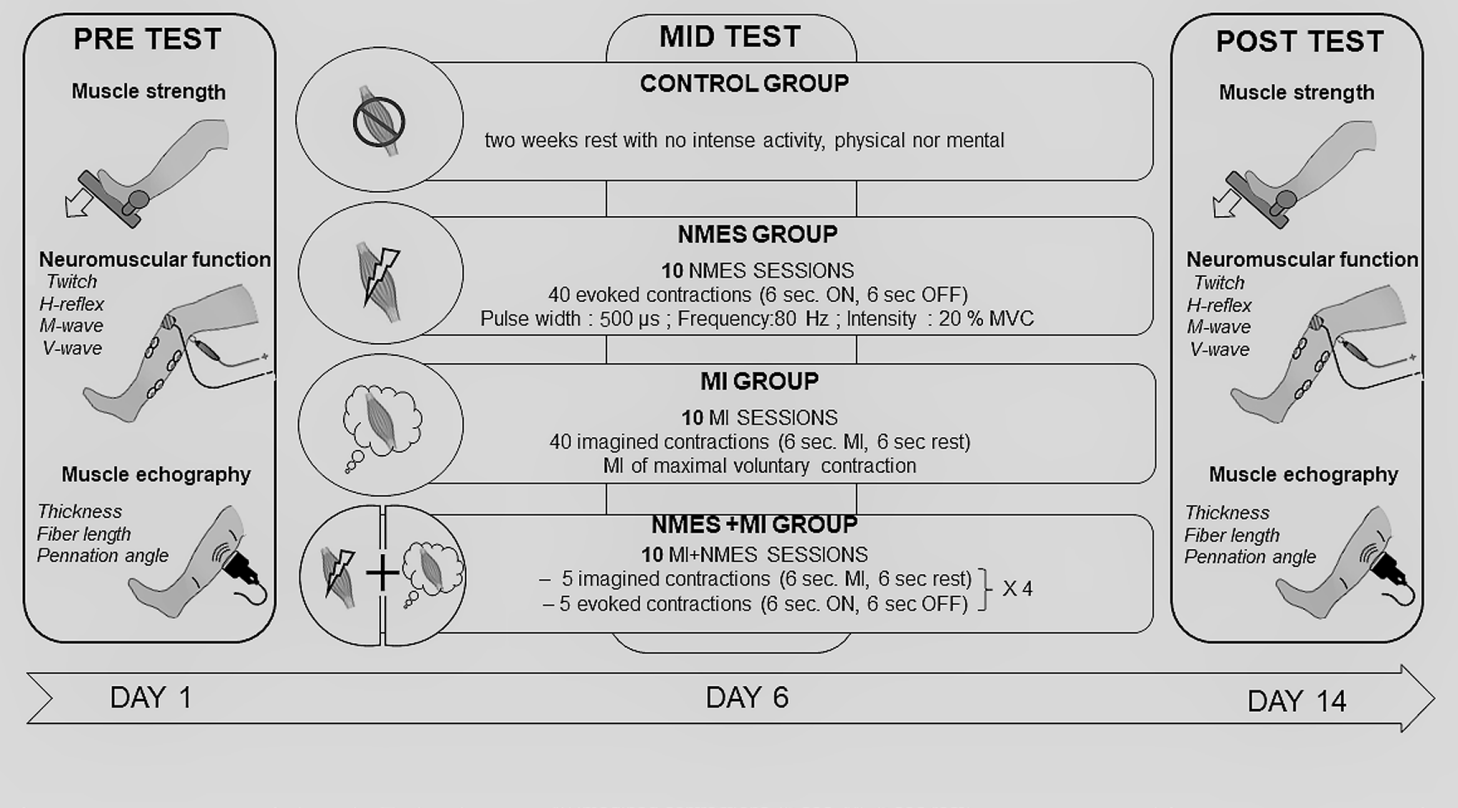
Overview of the experimental protocol

All participants performed a 2-week training on their right triceps surae preceded and followed by assessment of the neuromuscular function (Fig. 1). Participants were randomly distributed into 4 independent groups: control group (CON, *n* = 7, 2 females), neuromuscular electrical stimulation group (NMES, *n* = 10, 3 females), motor imagery group (MI, *n* = 10, 4 females) and combined group (NMES + MI, *n* = 10, 3 females) according to the training they would undergo on their right plantar flexor muscles. While the control group did not perform any exercise during the duration of the protocol, each of the other groups followed 10 training sessions (5 per week) which is in the range of those previously found in the literature: Sidaway and Trzaska (2005) used 12 sessions of motor imagery, Grosprêtre et al. (2017) used 7 sessions of motor imagery, Gondin et al. (2006) used 15 sessions of NMES. For the trained groups, measurements were performed 3 days before the first training session and the day after the last training session, respectively for PRE- and POST-tests. Participants were familiarized to MI technique and/or to NMES during the inclusion visit at the laboratory, and during the first experimental session.

The same neurophysiological variables were measured in each of the four groups PRE- and POST-training (Fig. 1).

The PRE and POST tests consisted in performing maximal voluntary isometric contractions (MVC), ultrasound measurements and neuromuscular assessments. The testing sessions lasted 1h30 in average and always took place in the laboratory between 9h30 and 16h30.

Each PRE and POST testing session started with muscle architecture assessment based on ultrasound measurements. During the rest of the testing session, participants seated on the chair of an isokinetic ergometer (Biodex system 4, Shirley, NY). The ergometer enabled instantaneous torque recording that was monitored throughout the experimental session. Electromyographic activities of the right triceps surae and tibialis anterior muscles were also recorded throughout the session.

Once installed, participants were asked to warm up their plantar flexor muscles by pushing sub-maximally against the pedal ten times with a force increment. Peripheral electrical nerve stimulations were sent to the posterior tibial nerve to elicit H-reflexes and M-waves of triceps surae.

### Training sessions

Participants in NMES, MI and NMES + MI performed a 2-week training (10 days) consisting of isometric contractions on their right plantar flexor muscles. According to their group, the training was based on NMES, motor imagery or a combination of both. Participants in the control group did not train during this period. Each training session lasted 20–30 min and was conducted mainly between 12 and 14 h in the laboratory. During each training session, participants seated on the chair of the ergometer (Biodex system 4, Shirley, NY) with their right foot firmly tied on a pedal and set at a 90°-ankle angle as during PRE and POST tests.

### NMES training sessions

Forty trains (6 s ON, 6 s OFF) of monophasic rectangular electrical stimuli were delivered to the triceps surae muscles by a high voltage (400 V) constant-current stimulator (Digitimer model DS7A, Hertfordshire, UK) triggered by a commercially available software (Tida, Heka Elektronik, Lambrecht/Pfalz, Germany). A pair of self-adhesive gel electrodes (10 × 5 cm, Medicompex SA, Ecublens, Switzerland) was placed over the gastrocnemii (~ 5 cm below the popliteal fossa) and soleus (~ 10 cm above the calcaneus) muscles. Their contours were traced with a permanent marker to ensure that electrodes were always placed in the same position. Each train consisted in 6 s of electrical pulses with duration fixed at 500 µs, evoked at a constant frequency of 80 Hz. Before each training session, pulse intensity was set to evoke an initial force level corresponding to 20% of the MVC (10–45 mA) using 3 testing trains of 2 s, adapted to each participant. This intensity was not modified during the session.

### MI training sessions

Each participant who practiced MI first completed the revised version of the Movement Imagery Questionnaire (MIQ-R Hall and Martin 1997) to determine self-estimation of MI ability. The initial mean MIQ-R score was 46.1 ± 6.1 (maximum score: 56), indicating a good imagery capacity in all participants. Participants performed each MI training session in the laboratory under the supervision of the same experimenter. MI training sessions consisted of 40 MI of maximal isometric plantar flexion of the right leg during 6 s, interspaced with a rest period of 6 s. Participants were instructed to imagine pushing maximally on the pedal of the ergometer, and to associate the mental representation of the sensations normally experienced during actual performance (kinesthetic modality). Each imagined trial was preceded and stopped by an auditory signal. The continuous recording of force signal and background electromyography (EMG) of triceps surae muscles helped to ensure that no motor output was provided during the completion of the MI task and that participants stayed fully relaxed. Participants were asked to rate the quality of their MI training session with a quote from 1 (poor) to 10 (excellent). During the training, the self-estimated imagery (mean: 7 ± 2 out of 10) did not vary significantly.

### Combined MI + NMES training sessions

The NMES + MI group underwent 20 MI and 20 evoked contractions for a total of 40 contractions like the other training programs. These contractions were implemented in 4 blocks of 5 evoked contractions (6 s ON and 6 s OFF) and 5 imagined contractions (6 s MI and 6 s rest) in this order. Each MI and NMES trials were performed with the same modality of single NMES and MI training described above. The initial mean MIQ-R score was 44.1 ± 5.4 (maximum score: 56). During training, self-estimated imagery (mean: 7 ± 2 out of 10) did not vary significantly and the stimulation intensity range was 10–45 mA, adapted to each participant and reassessed at the beginning of each training session to match 20% MVC.

In both NMES and NMES + MI groups, MVC was reassessed during the first and the sixth sessions to readjust NMES intensity to still correspond to the same percentage of the actualized MVC performance. To standardize the protocol, MI and CONTROL groups also performed 2 MVC during the first and the sixth sessions. Before these two training sessions, participants warmed-up by pushing sub-maximally 10 times against the pedal, then performed 2 maximal isometric contractions in the same conditions as PRE and POST tests.

### Mechanical recordings

The torque of the participant during each actual and evoked contraction was measured by means of an isokinetic dynamometer (Biodex system 4, Shirley, NY). The axis of the dynamometer was aligned with the right external malleolus. Subjects were placed with the hip and knee joints at 90° (180° = full extension), and ankle joint at 90° (angle between the leg and the sole). During all conditions, care was taken to avoid trunk and head rotations to maintain constant corticovestibular influences on the excitability of the motor pool (Schieppati 1987). The foot was firmly strapped to the pedal of the dynamometer in two places, i.e. the end of the metatarsus and the junction with the shinbone, to prevent the heel from lifting. The trunk was stabilized by two crossover shoulder harnesses. Torque signal was recorded continuously during the whole protocol.

### Electromyography (EMG) recording

EMG activity was recorded from four muscles of the leg (soleus: SOL, gastrocnemius medialis: GM, gastrocnemius lateralis: GL; tibialis anterior: TA). After shaving and dry-cleaning the skin with alcohol to keep a low impedance (< 5 kΩ), EMG signals were recorded using two silver chloride surface electrodes (8 mm diameter) placed at an interelectrode center-to-center distance of 2 cm. For SOL, electrodes were placed 2 cm below the insertions of the gastrocnemii over the Achille’s tendon; for GM and GL, over the mid belly of the muscles; and for TA, at 1/3 of the distance between the fibula and the lateral malleolus. A common reference electrode was placed between stimulation and recording sites on the lower part of the belly of the gastrocnemii. EMG signals were amplified with a bandwidth frequency ranging from 15 Hz to 1 kHz (gain = 1000) then digitized online (sampling frequency: 5 kHz) using the MP150 Biopac system and stored for analysis with Acqknowledge software 4.2.

### Muscle architecture

Participants lay in a static prone position for 10 min before any measure was taken. Then, a 5.5-cm (7.5 MHz) linear array probe was positioned perpendicular to the dermal surface and oriented along the longitudinal axis of the muscle–tendon unit. The GL upper insertion was found in the popliteal fossa and marked. A measure was taken from this mark to reach the belly of the GL and transferred to the GM as well. This distance (10–14 cm) was noted on the skin with a permanent marker for POST tests. Three images of each muscle were recorded using B-mode Zonare ultrasound video imaging (Z. One ultra sp 4.2, Zonare Medical Systems Inc. Mountain View, CA, USA). Fascicle length (FL), pennation angle (Pα) and muscle thickness (MT) were extracted. Criteria for storing images were parallel superficial and deep aponeurosis, and the presence of at least three discernible fascicles with their junction upon deep aponeurosis. On each image, MT was measured from the average distance between the deep and the superficial aponeurosis measured directly by the ultrasound software. Pα and FL were calculated.

### Neuromuscular assessment

Right posterior tibial nerve percutaneous nerve stimulations were used to record H-reflexes and M-waves of the considered muscles. Single rectangular pulses (1-ms width) were delivered by a high voltage (400 V) constant-current stimulator, through a self-adhesive anode (8-mm diameter, Ag–AgCL) placed in the popliteal fossa and a cathode (5 × 10 cm, Medicompex SA, Ecublens, Switzerland) placed over the patella. Optimal stimulation site was first located by a hand-held ball electrode (0.5-cm diameter) to obtain the greatest H-reflex amplitudes for the lowest stimulation intensity, focusing on the SOL muscle. Particular care was taken in avoiding EMG responses on the antagonist muscle (TA). Once determined, stimulation electrodes were firmly fixed to the optimal site with straps.

To build H–M recruitment curves, stimulations started at the H-reflex threshold and were progressively increased with a 4 mA increment until M-wave amplitude no longer increased. To ensure that the M-wave lay in the plateau of its maximal value, maximal stimulation intensity was increased by 20% and taken as *M*_MAX_ intensity. Three responses were evoked at each intensity, with an inter-stimulus interval of 10 s. Any response with a voluntary pre-stimulus was removed. The whole curve from H-reflex threshold to *M*_MAX_ was performed PRE and POST training.

The intensity of the maximal H-reflex, noted *H*_MAX_ at rest and *H*_SUP_ during contraction, was used to assess spinal excitability during maximal contractions (MVC). As well, superimposed twitches at *M*_MAX_ intensity were evoked during MVC of the plantar flexor muscles. Contrary to rest, it can be noticed that the evoked maximal M-wave (*M*_SUP_) is accompanied by a reflex wave noted V-wave, which is an electrophysiological variant of the H-reflex virgules noter. Stimulation were manually triggered once the torque lay in the plateau of its maximal value. Participants were asked to maintain the contraction for 1 s after the stimulation. Two stimulations were performed for *H*_SUP_ and two for *M*_SUP_/V-wave during separated MVC, with at least 1-min rest between.

## Data analysis

### Mechanical responses

The maximal force was measured as the peak of the torque developed during MVCs when the signal lay in the plateau of its maximal value. Within subjects data were carefully checked for extremes values i.e., different from more than two standard deviations of the mean. Therefore, over the six to eight trials performed for MVC measurements at each time point (PRE and POST), 2 values of 1 participant in the MI group and 2 values of 2 participants in the NMES group were discarded. These discarded trials, showing abnormally high MVC values, were attributed to a high recruitment of the quadriceps and/or the gluteal muscles during the completion of the plantar flexion, therefore compensating with the triceps surae muscle recruitment. It should be noticed that this correction has been performed for MVC only, other data not showing such extreme values. Delta values of strength gains were calculated as (POST-values − PRE-values)/PRE-values × 100.

Mechanical twitches following maximal M-wave stimulations at rest were also analyzed to account for peripheral changes*.* Twitches were measured as the peak amplitude of the torque signal. The electromechanical efficiency (EME) was then calculated by the ratio of this peak twitch (Pt) over the sum of amplitudes of the three corresponding maximal M-waves, i.e., SOL, GM and GL (Pt/*M*_MAX_). For NMES and NMES + MI, the area under the curve of each electrically evoked contraction, i.e., the torque-time integral (TTI), was determined during all training sessions. The TTI of all evoked contractions was summed for each training session in NMES and NMES + MI groups to express the total TTI.

### Electrophysiological responses

Peak-to-peak amplitudes of each EMG response at rest and superimposed to MVC (M-waves, H-reflexes, V-waves) were measured and averaged among the different trials. The amplitude of the submaximal M-wave accompanying each H-reflex, noted *M*_atH_, was also considered.

Rest recruitment curves of H- and M-waves were built for each muscle of the triceps surae by plotting stimulus intensities against response amplitudes. All H-reflexes and submaximal M-wave values were normalized by the corresponding maximal M-wave. As well, the intensity necessary to obtain each response was expressed as a percentage of the maximal intensity (i.e., *M*_MAX_ intensity). Mean recruitment curves over all participants were built by sorting out the *H*–*M* values by ranges of 10% of *M*_MAX_ intensity. The average of all the participants in each range was used for statistical analysis performed on the recruitment curves.

RMS was measured during MVCs and normalized by maximal M-wave amplitude measured during MVC (*M*_SUP_).

### Muscle architecture

For each subject, three images of GM and GL muscles were recorded. On each of them, we measured three architectural parameters with the ultrasound software. The first one was muscle thickness taken between the deep and the superficial aponeurosis, perpendicularly to the deep aponeurosis, on the left and right edges, and at the middle of the image. The second measure called d1 was the partial fascicle length. Indeed, for most of the subjects, muscle fascicles could not be directly measured by the software since fascicles were longer than the screen of the ultrasound device. The third measure called d2 was the distance between the deep aponeurosis and the end of the partial fascicle, taken perpendicularly to the deep aponeurosis. Thus, d1 and d2 designed a squared triangle with the deep aponeurosis, d1 being the hypotenuse. From these measurements, pennation angle and fascicle length were obtained by calculating Pα = arcsin (d2/d1) and FL = muscle thickness/sin (Pα), respectively. At last, three measurements from the three images were averaged for each subject and each muscle.

### Statistical analysis

All data are presented as the mean ± standard deviation. Shapiro–Wilk test (*p* < 0.05) was used to ensure the normality and homogeneity was assessed by means of Levene test. Each muscle was assessed separately. A two-way ANOVA with repeated measures was performed on each dependent variable with the factor *group* (CONTROL, MI, NMES, and NMES + MI) and the factor *time* (PRE and POST). When a main or interaction effect was found, a post-hoc analysis was conducted using Tukey’s HSD (honest significant difference) test. Statistical analysis was performed with Statistica software (10.0 version, Statsoft, Tulsa, Oklahoma, USA). The level of significance was set at *p* < 0.05.

## Results

### Strength

**Fig. 2:**
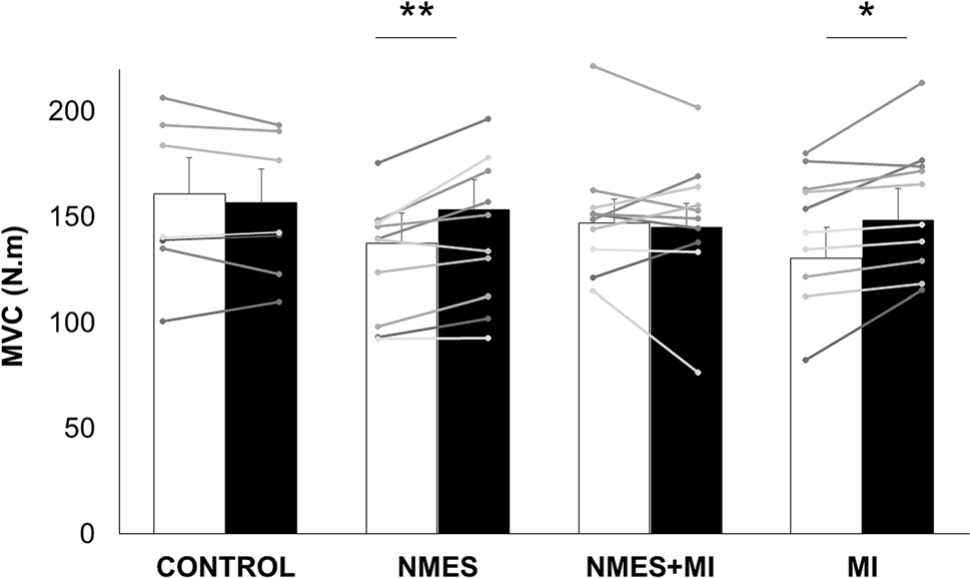
Plantar flexor muscles force characteristics of the four groups. Data are depicted as mean ± SD for the four groups: the control group (CONTROL) and the three trained groups: neuromuscular electrical stimulation only (NMES), combined NMES and motor imagery (NMES + MI) and MI only (MI). Maximal torque of the plantar flexor muscles recorded during maximal voluntary contraction (MVC) before (PRE, white bars) and after (POST, black bars) the training protocols. Individual data are depicted in gray dots and lines. *,**Significant PRE-POST difference at *p* < 0.05 and *p* < 0.01, respectively

A significant *time* × *group* interaction was found for the maximal torque (*F*_3,33_ = 4.129, *p* = 0.013). Pre-values were not statistically different between groups (all *p* > 0.53). The maximal torque was significantly increased by 11.3 ± 3.5% for the NMES group and by 13.8 ± 5.6% for the MI group (Fig. 2a). A statistical analysis of these PRE–POST deltas revealed a significant effect of the factor *group* (*F*_3,33_ = 12.42, *p* < 0.001). PRE-to-POST changes were significantly higher in NMES and MI groups as compared to CONTROL (*p* < 0.001 and *p* = 0.0023, respectively) and NMES + MI (*p* = 0.004 and *p* = 0.002, respectively). There was no difference between NMES and MI (*p* = 0.934).

For the NMES group, the total TTI among the ten training sessions was 20 423.2 ± 6 794.8 Nm s. The averaged total TTI for the NMES + MI group was significantly lower (17 441.6 ± 8 990.4 Nm s, *p* = 0.003).

### Muscular parameters

No main effect or interaction was found for any of the architectural parameters measured by ultrasound, such as MT, Pα or FL (Table 1). No parameter was modified after any of the tested training or for the control group.

**Table 1:** Ultrasound data including muscle thickness (cm), pennation angle (°) and fascicle length (cm)

	Thickness		Pennation angle		Fiber length	
	PRE	POST	PRE	POST	PRE	POST
Gastrocnemius medialis						
MI	1.8 ± 0.3	1.8 ± 0.3	19.9 ± 2.3	21.2 ± 6.4	5.5 ± 0.8	5.3 ± 1.1
NMES	2.0 ± 0.3	2.0 ± 0.2	19.5 ± 2.7	20.8 ± 2.6	5.9 ± 0.7	5.6 ± 0.6
NMES + MI	1.8 ± 0.2	1.7 ± 0.3	20.9 ± 3.7	19.2 ± 3.5	5.3 ± 0.8	5.3 ± 0.9
CONTROL	2.1 ± 0.2	2.1 ± 0.3	23.0 ± 3.8	20.9 ± 3.4	5.6 ± 1.1	6.0 ± 1.1
Gastronemius lateralis						
MI	1.4 ± 0.3	1.4 ± 0.2	13.3 ± 2.0	14.7 ± 2.4	6.1 ± 1.5	5.7 ± 0.7
NMES	1.5 ± 0.3	1.5 ± 0.3	14.4 ± 3.2	13.9 ± 2.1	6.0 ± 0.9	6.3 ± 0.5
NMES + MI	1.4 ± 0.4	1.3 ± 0.3	13.2 ± 4.3	12.0 ± 2.9	6.3 ± 1.6	6.2 ± 1.7
CONTROL	1.5 ± 0.2	1.4 ± 0.2	12.7 ± 2.9	11.5 ± 2.7	7.1 ± 2.0	7.2 ± 1.8

On the contrary, a significant *time* × *group* interaction was found for the plantar flexor muscles peak twitch associated with supramaximal stimulation (*F*_3,33_ = 3.82, *p* = 0.019). The peak twitch was increased from PRE to POST only after NMES training (*p* = 0.011), from 21.9 ± 7.9 to 26.5 ± 8.9 N m, and remained unchanged for the three other groups (CONTROL: from 24.5 ± 7.2 to 25.5 ± 8.5 N m; MI: from 20.8 ± 8.9 to 22.4 ± 6.3 N m; NMES + MI: from 21.6 ± 7.7 to 21.2 ± 6.6 N m). A significant *time* × *group* interaction was found for maximal muscle action potential, i.e. *M*_MAX_ amplitude, for SOL muscle only (*F*_3,33_ = 4.353, *p* = 0.011). SOL maximal M-wave was increased after NMES training only (*p* = 0.018), from 12.3 ± 3.7 to 14.8 ± 3.7 mV (Table 2), while no change has been found in the three other groups. As well, a significant increase in EME has been found for NMES group only (*p* = 0.009), from 0.76 ± 0.27 to 0.89 ± 0.29 N m/mV (CONTROL: from 0.82 ± 0.2 to 0.81 ± 0.2 N m/mV; MI: from 0.70 ± 0.3 to 0.73 ± 0.3 N m/mV; NMES + MI: from 0.82 ± 0.3 to 0.83 ± 0.3 N m/mV).

**Table 2:** Mean ± SD raw values for the four groups (mV)

	CONTROL		NMES		NMES + MI		MI	
	PRE	POST	PRE	POST	PRE	POST	PRE	POST
SOL								
*H*_MAX_	4.4 ± 1.6	4.5 ± 2.0	4.7 ± 2.1	3.3 ± 2.1	4.0 ± 2.5	4.7 ± 2.2	3.4 ± 1.5	4.6 ± 2.6
*M*_atHmax_	2.2 ± 1.1	2.0 ± 1.1	2.1 ± 1.2	4.0 ± 0.8	2.1 ± 1.6	2.1 ± 1.4	2.1 ± 1.5	2.1 ± 1.4
*M*_MAX_	9.8 ± 4.0	10.4 ± 1.8	12.3 ± 3.7	14.8 ± 3.7	11.4 ± 4.5	11.1 ± 4.4	10.6 ± 4.7	10.9 ± 3.4
*H*_SUP_	5.9 ± 1.4	6.3 ± 1.1	6.7 ± 3.3	6.0 ± 1.7	5.7 ± 2.9	5.2 ± 3.6	7.1 ± 3.7	6.9 ± 5.1
*M*_atHsup_	2.1 ± 2.3	1.6 ± 0.9	3.7 ± 2.5	3.0 ± 2.4	1.2 ± 1.0	1.8 ± 1.7	3.8 ± 2.4	3.2 ± 2.9
*M*_SUP_	12.1 ± 3.3	12.7 ± 2.2	12.0 ± 3.7	12.8 ± 3.7	13.0 ± 4.0	12.9 ± 3.0	13.3 ± 3.0	13.5 ± 2.9
*V*	4.6 ± 1.0	3.9 ± 0.7	3.6 ± 1.9	5.7 ± 2.0	3.4 ± 1.8	4.6 ± 2.9	3.0 ± 2.9	6.5 ± 4.1
GM								
*H*_MAX_	1.5 ± 0.7	1.9 ± 1.0	1.7 ± 1.2	1.2 ± 1.0	1.1 ± 0.8	1.2 ± 0.7	1.3 ± 1.1	2.1 ± 0.8
*M*_atHmax_	3.4 ± 1.5	3.3 ± 2.1	4.3 ± 2.4	6.4 ± 3.0	3.5 ± 1.1	3.6 ± 2.5	3.1 ± 2.0	3.1 ± 2.3
*M*_MAX_	5.7 ± 2.0	5.9 ± 2.1	6.8 ± 1.9	7.3 ± 2.8	6.1 ± 4.4	5.3 ± 2.0	7.1 ± 4.7	6.7 ± 3.4
*H*_SUP_	3.1 ± 1.1	3.2 ± 1.6	3.5 ± 1.9	3.6 ± 1.6	2.4 ± 1.2	2.1 ± 1.1	3.4 ± 2.3	3.2 ± 1.8
*M*_atHsup_	2.3 ± 1.7	3.2 ± 2.2	3.60 ± 3.0	4.0 ± 4.1	1.7 ± 1.3	2.6 ± 1.7	4.9 ± 3.8	4.1 ± 3.2
*M*_SUP_	8.8 ± 3.0	8.2 ± 2.9	10.3 ± 4.6	10.8 ± 4.1	7.3 ± 4.5	6.4 ± 3.7	9.8 ± 4.5	9.5 ± 3.3
*V*	2.1 ± 1.1	1.8 ± 1.1	1.8 ± 1.4	2.7 ± 1.2	2.1 ± 0.5	2.4 ± 0.6	2.0 ± 2.3	3.2 ± 1.6
GL								
*H*_MAX_	1.3 ± 0.5	1.6 ± 0.8	2.0 ± 2.1	1.0 ± 1.0	1.3 ± 0.9	1.3 ± 0.9	1.2 ± 0.8	1.6 ± 0.8
*M*_atHmax_	3.2 ± 2.7	4.7 ± 2.9	4.0 ± 3.9	5.3 ± 3.4	4.3 ± 4.4	4.1 ± 3.3	5.6 ± 3.5	5.6 ± 4.3
*M*_MAX_	8.0 ± 1.6	8.4 ± 0.7	10.8 ± 4.0	8.8 ± 2.6	10.3 ± 5.4	9.0 ± 4.1	8.8 ± 3.2	9.2 ± 3.1
*H*_SUP_	2.8 ± 0.8	2.8 ± 1.2	3.5 ± 2.7	3.0 ± 1.7	3.2 ± 2.8	2.7 ± 2.4	2.1 ± 1.3	2.5 ± 1.5
*M*_atHsup_	2.6 ± 3.0	2.9 ± 2.8	3.6 ± 3.1	3.0 ± 1.2	2.9 ± 2.0	3.3 ± 2.9	3.1 ± 2.0	3.9 ± 2.9
*M*_SUP_	12.1 ± 3.3	10.5 ± 2.6	12.7 ± 4.2	10.1 ± 3.2	13.0 ± 4.0	11.5 ± 3.3	12.7 ± 3.7	11.0 ± 3.9
*V*	1.7 ± 0.7	1.8 ± 1.0	1.9 ± 1.5	3.1 ± 1.3	1.9 ± 1.5	1.5 ± 0.7	2.0 ± 1.8	3.0 ± 1.3

### Nervous parameters

**Fig. 3:**
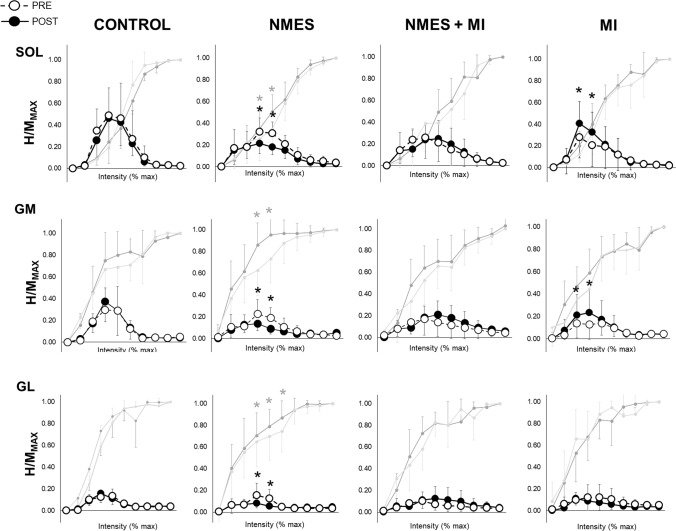
Spinal excitability of the plantar flexor muscles in the four groups. Spinal excitability is expressed as the recruitment curves the H-reflexes and M-waves of the soleus (SOL), gastrocnemius medialis (GM) and gastrocnemius lateralis (GL) recorded for the four groups (CONTROL; *NMES* neuromuscular electrical stimulation only, *MI* motor imagery, *NMES + MI* combined training). Recruitment curves are built as follows: mean H-reflex responses are normalized by the corresponding maximal M-wave (*M*_MAX_) in ten portions of intensities (portion 1: from 0 to 10% of maximal intensity; portion 2: from 11 to 20% of maximal intensity; …; portion 10: from 91 to 100% of maximal intensity). Open circles are responses recorded at PRE, and full circles are responses recorded at POST. Associated M-waves to each H-reflex, also normalized by the corresponding maximal M-wave, are represented in light gray for PRE and dark gray for POST. *Significant PRE-to-POST response at *p* < 0.05 (dark: for *H*/*M*_MAX_ and gray for *M*_atH_/*M*_MAX_)

Regarding the recruitment curves, while no change was observed either in the control or in the NMES + MI group, some PRE-POST differences have been found in the two other groups (Fig. 3).

In the NMES group, a significant decrease in *H*/*M*_MAX_ ratios including *H*_MAX_/*M*_MAX_ has been observed in the three muscles from PRE to POST at 40% (SOL: *p* = 0.025; GM: *p* = 0.016; GL: *p* = 0.047) and 50% of maximal intensity (SOL: *p* = 0.020; GM: *p* = 0.028; GL: *p* = 0.045). The associated *M*_atH_/*M*_MAX_ were significantly higher in POST than in PRE for those two intensities (40%: SOL: *p* = 0.022; GM: p = 0.036; GL: *p* = 0.044; 50%: SOL: *p* = 0.016; GM: *p* = 0.035; GL: *p* = 0.022). This PRE-to-POST decrease of *H*/*M*_MAX_ ratios at 40 and 50% of maximal intensity was significantly correlated to the increase of *M*_atH_/*M*_MAX_ ratios at the same ranges of intensities (SOL: *r* = 0.96; GM: *r* = 0.87; GL: *r* = 0.71).

In the MI group, a significant PRE-to-POST increase in *H*/*M*_MAX_ ratios including *H*_MAX_/*M*_MAX_ was observed at 30% and 40% of maximal intensity in SOL (30%: *p* = 0.041 and 40%: *p* = 0.023) and in GM (30%: *p* = 0.033 and 40%: *p* = 0.018), with no change in *M*_atH_/*M*_MAX_.

**Fig. 4:**
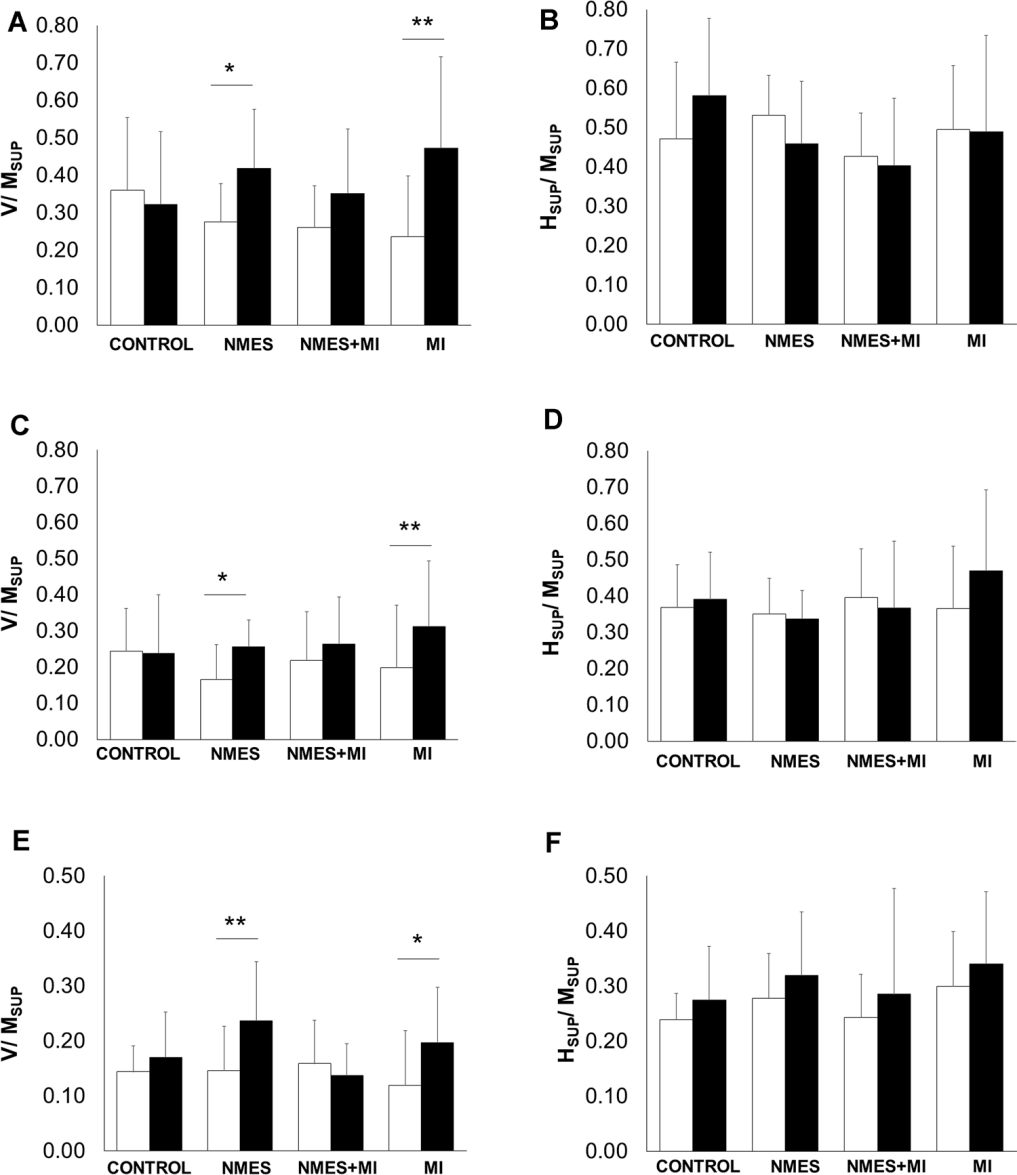
Plantar flexors V-wave and superimposed H-reflex of the four groups. *V*/*M*_SUP_ and *H*_SUP_/*M*_SUP_ ratios are depicted for the four groups: the control group (CONTROL) and the three trained groups: neuromuscular electrical stimulation only (NMES), combined NMES and motor imagery (NMES + MI) and MI only (MI). Ratios before (PRE, white bars) and after (POST, black bars) are shown for the three tested muscles in **a** and **b** for soleus muscle, **c** and **d** for gastrocnemius medialis, and **e** and **f** for gastrocnemius lateralis. *,**Significant PRE-POST difference at *p* < 0.05 and *p* < 0.01, respectively

A significant *time* × *group* interaction has been found for *V*/*M*_SUP_ in SOL (*F*_3,33_ = 4.465, *p* = 0.010), in GM (*F*_3,33_ = 3.562, *p* = 0.024) and in GL (*F*_3,33_ = 5.114, *p* = 0.005). As depicted in Fig. 4*V*/*M*_SUP_ ratios were significantly enhanced after NMES training (SOL: *p* = 0.012; GM: *p* = 0.474; GL: *p* = 0.007) and after MI training (SOL: *p* = 0.004; GM: *p* = 0.002; GL: *p* = 0.033).

No significant main effect or interaction was found for SOL superimposed H-reflex, i.e. *H*_SUP_/*M*_SUP_ (*F*_3,33_ = 1.129, *p* = 0.352), neither for GM (*F*_3,33_ = 1.066, *p* = 0.377) nor GL (*F*_3,33_ = 0.226, *p* = 0.878).

A significant interaction was found on muscular activity recorded during MVC (RMS/*M*_SUP_), for SOL (*F*_3,33_ = 5.175, *p* = 0.005), GM (*F*_3,33_ = 5.943, *p* = 0.002) and GL (*F*_3,33_ = 3.291, *p* = 0.033). RMS/*M*_SUP_ was increased after NMES and MI training for SOL (*p* = 0.003 and *p* = 0.020, respectively), GM (*p* = 0.010 and *p* = 0.013) and GL (*p* = 0.035 and *p* = 0.019).

## Discussion

The present study investigated the effect of a 2-week intervention program on plantar flexor muscles with alternated NMES + MI compared to MI and NMES alone. The maximal performance, i.e. MVC, only increased for MI and NMES groups while no change has been found in the combined NMES + MI group. This was accompanied with supraspinal changes, as evidenced by an increased *V*/*M*_SUP_ in SOL, GM and GL for both NMES and MI groups without any modifications of *H*_SUP_/*M*_SUP_. Spinal plasticity at rest occurred for both MI and NMES groups but in different directions. While a decrease in *H*/*M*_MAX_ was observed in the NMES group, an increase in *H*/*M*_MAX_ was found in SOL and GM in the MI group. No change in muscle architecture has been found in any group. The combined group did not exhibit either neural or muscular changes.

### NMES training alone

In the present study, significant strength gains were observed in the NMES group with an increase in MVC by 11.3 ± 3.5% after 2 weeks of training at a targeted force of 20% MVC. Changes at central level were observed, whereas NMES training did not involve changes in muscle architecture.

Strength gains following NMES training are widely reported in the literature. Despite different methodologies in terms of stimulation site, targeted evoked force, pulse width and frequencies, strength gains vary from 8 to 22% for plantar flexors (Maffiuletti et al. 2001; Gondin et al. 2006). Similar strength gains have been reported by Gondin and colleagues in 2006 (Gondin et al. 2006) and Maffiuletti and colleagues in 2001 (Maffiuletti et al. 2001) following NMES training induced by maximal tolerable stimulation intensity, leading to considerable evoked force by subjects throughout each session (between 50 and 70% MVC for both studies) contrary to the present study. Therefore, it seems that NMES training is an effective stimulus for strength development even when the level of evoked force during training is relatively low.

In the present study, no architectural changes have been observed, suggesting that the short training period associated with low levels of evoked force does not induce a sufficient mechanical stress. Indeed, this adaptation has been obtained after longer NMES training periods using higher stimulation intensities (Gondin et al. 2005; Lee and Yoon 2009). Nevertheless, some other changes at muscular levels were still observed.

Indeed, the peak twitch (Pt) and SOL *M*_MAX_ were increased from PRE to POST NMES training while GM *M*_MAX_ and GL *M*_MAX_ remained unchanged. An increase in Pt after NMES has already been shown in a few studies (Maffiuletti et al. 2003; Colson et al. 2009). Taken together, those changes led to a significant increase in electromechanical efficiency ratio (EME) found for NMES group only, indicating an improvement in excitation/contraction coupling efficiency. This parameter reflects the entire sequence of reactions from the spread of the action potential across the neuromuscular junction to the release of Ca^2+^ and the cross-bridge interaction (Sandow 1952; Melzer et al. 1995). Thus, NMES training may have induced a more important release in calcium and/or a higher calcium sensitivity of contractile proteins. Through percutaneous muscle biopsies taken from the vastus lateralis muscle, a study of Russ and colleagues (2012) (Russ et al. 2012) showed that after NMES training (30% MVC, 80 Hz for 5 weeks) the peak rate of Ca^2+^ release was significantly enhanced (~ 16%) (Russ et al. 2012). The lack of structural changes highlighted that peak twitch and EME evolution after NMES training rather originated from muscle intracellular processes.

It should be noticed that, in addition to an improvement in excitation/contraction coupling efficiency, higher nervous levels seemed involved in the strength gains observed after NMES training. NMES evokes action potentials in both intramuscular nerve branches (Hultman et al. 1983) and cutaneous receptors, thus also resulting in reflexive recruitment of spinal motoneurons (Collins et al. 2001). In the present study, since *H*_SUP_/*M*_SUP_ ratio of all three muscles was unaffected, spinal modulation seemed poorly involved in the strength gains associated with NMES.

Although spinal excitability during MVC remained unchanged, it decreased at rest following NMES training. This decrease was accompanied by an increase in submaximal M-waves associated with H-reflexes, i.e. *M*_atH_, in all muscles (Fig. 3). Such an enhancement of *M*_atH_ can induce an increase in antidromic current elicited by electrical impulses generated in motor axons by nerve stimulation (Piscione et al. 2012). This may have therefore canceled the reflexive activation, leading to lower H-reflexes at 40 and 50% *M*_MAX_ intensity in POST tests. At these intensities, the positive correlation between such H-reflex depression and the increase in *M*_atH_/*M*_MAX_ supports this hypothesis. It emphasizes the fact that the so-observed decrease in *H*/*M*_MAX_ may not necessarily be due to a depression of spinal excitability per se. NMES leads to both direct and indirect activation of motor axons toward the muscle (Trimble and Enoka 1991). Here, the direct and repetitive activation during training may have caused an increase in axonal excitability leading, for submaximal nerve stimulation intensities, to a higher number of recruited motor units. Nevertheless, the measure of H-reflex does not necessarily reflect all the structures acting at the spinal level. An increase in spinal presynaptic inhibition following acute NMES training has been previously observed (Grosprêtre et al. 2018). Such an adaptation could also play a role in the decrease in rest H-reflex following NMES training.

However, NMES afferent or indirect activation led to plasticity of other nervous levels, as interpreted from the results of *V*/*M*_SUP_ and *H*_SUP_/*M*_SUP_. Measured during voluntary contraction, the V-wave reflects motoneuron activation through afferents which benefited from the collision occurring between orthodromic motor action potentials elicited by the descending voluntary input to the motoneuron pool and the antidromic current generated in the motor axons by the external stimulation (Aagaard et al. 2002). Therefore, it is assumed that the magnitude of the collision is related to the descending motor command (Grosprêtre and Martin 2014). Nonetheless, by being a reflexive response, V-wave amplitude also involves motoneuron responsiveness (e.g., changes of intrinsic membrane properties and discharge rate), synaptic transmission efficacy at Ia afferent terminals (e.g., presynaptic inhibition), and/or postsynaptic inhibition (Carroll et al. 2011). These mechanisms are usually ruled out by measuring also the superimposed H-reflex to the same level of contraction, i.e., *H*_SUP_. Consequently, it has been assumed that in the absence of any *H*_SUP_ modulation, the increase of V-wave amplitude reflects an increase of supraspinal activation, which enhances descending volitional drive (i.e., recruitment and/or motoneuron firing frequency) to the muscle (Aagaard et al. 2002), as it can be observed in the present NMES training. Such results have already been observed after 15 sessions of NMES applied over the triceps surae (Gondin et al. 2006), where the change in V-wave amplitude has been attributed to the cortical influence of NMES. To corroborate this hypothesis, previous works have already shown by functional neuroimaging that premotor and primary motor cortices can be activated during NMES (Han et al. 2003). It has been postulated that the brain activation induced by NMES, although not being systematically associated with changes at spinal level, might then imply a larger loop involving the sensory motor cortex and the motor cortex, both being interconnected. Such supraspinal action of NMES might then be one of the main mechanisms involved in such an increase in V-wave.

### MI training alone

In the present study, MI training group also increased their plantar flexor muscles MVC by 13.8 ± 5.6%. Although training protocols vary, strength gains of a higher magnitude have already been reported with MI modality (e.g., Yue and Cole 1992: + 22% after 4 weeks; Zijdewind et al. 2003: + 36% after 7 weeks and Grosprêtre et al. 2017: + 9.6% after 1 week). Contrary to NMES, this gain has been attributed solely to neural adaptations rather than muscular changes (Ranganathan et al. 2004). In the present study, the lack of changes in muscle architecture and any parameters related to the excitation/contraction coupling (M-waves, twitches) following MI training also argue for a pure nervous plasticity.

At first, cortical reorganization, supposedly leading to an optimization of the central command and/or a better coordination of muscle activation (Ranganathan et al. 2004) has been proposed as the main mechanism involved. The increase in *V*/*M*_SUP_ ratio in SOL, GM and GL found in this study, without change in superimposed H-reflex (*H*_SUP_) after MI training also suggests a supraspinal adaptation. In line with preceding arguments regarding NMES-induced plasticity, such an increase in V-wave might occur because MI involves a particular activation of premotor and primary motor cortices (Decety et al. 1994). The increase of the motor evoked potential by transcranial magnetic stimulation observed in previous literature also argues for an increased excitability of the pathway from brain motor regions to the muscle (Grosprêtre et al. 2016). A previous work already showed that one week of daily MI practice led to an increase in triceps surae V-wave amplitude (Grosprêtre et al. 2017), emphasizing the benefits of MI to improve the ability to produce a great cortical descending command. However, in this last work such as in the present study, the measurement of rest spinal excitability PRE- and POST-MI training highlighted the involvement of another possible mechanism that implies spinal networks in the so-observed strength gains.

Noteworthy, significantly higher rest *H*/*M*_MAX_ ratios in both SOL and GM muscles were observed at 30% and 40% *M*_MAX_ intensity in POST tests, revealing an increase in rest spinal excitability after MI training. The lack of change in GL muscle could indicate that not all muscles have the same sensitivity to MI-induced plasticity. However, it should be pointed out that *H*/*M*_MAX_ ratios of GL muscles were low with a high variability, also explaining a lack of statistical changes with MI training despite low PRE-to-POST *p* values (*p* = 0.11). Regarding SOL and GM, the increase in *H*/*M*_MAX_ ratio could not be the result of changes in stimulation conditions onto the posterior tibial nerve since no change in M-waves accompanying H-reflexes (*M*_atH_/*M*_MAX_) was observed on both muscles (Grosprêtre and Martin 2012). Combined with the lack of change of superimposed H-reflex (*H*_SUP_/*M*_SUP_), these higher *H*/*M*_MAX_ ratios indicated that MI did not involve a global arousal of spinal circuitry but has impacted specific circuits. Indeed, while *H*_SUP_ involves both PRE- and POST-synaptic mechanisms, rest H-reflex changes are more likely attributed to presynaptic mechanisms at the level of Ia afferent-to-alpha motoneuron synapse (Zehr 2002; Misiaszek 2003) and to the excitability of the motoneuron itself (Grosprêtre et al. 2017). It is suggested that either the low-threshold motoneurons have become more excitable or the synapses of the Ia primary afferents became more efficient (Piscione et al. 2012). Indeed, MI was recently shown to result in a sub-threshold cortical motor output that could modulate the activity of spinal structures that mediates presynaptic inhibition of Ia terminal onto alpha motoneurons, i.e., the primary afferent depolarization interneurons (Grosprêtre et al. 2019). Overall, this put in evidence that such a partial activation of spinal circuitry during MI, when repeated in a training-designed protocol with multiple sessions of MI practice, might lead to a global increase in rest spinal excitability. Therefore, the neural adaptations accompanying the observed strength gains involved a larger part of the corticospinal system than usually expected, including both supraspinal and spinal plasticity.

### Combined NMES + MI training

Surprisingly, the combination of MI and NMES in the present study did not lead to observe any increase in performance, and in any of the peripheral and central factors measured, contrary to either modality performed alone. This lack of effect of NMES + MI is possibly explained by two hypotheses.

First, to provide the same training volume (total number of contractions) it should be reminded that the number of contractions was reduced for both NMES and MI in this group, as compared to NMES and MI groups. Therefore, half of the NMES program and half of the MI program have been performed in the NMES + MI group. Both may then represent an insufficient stimulus, at muscle level for NMES and nervous level for MI, to provide significant gains. This should indicate that gains in NMES and in MI do not involve the same mechanisms and that performing half of each does not lead to 100% or more of the NMES or MI gains. A longer training program with this design might reveal strength gains, arguing in favor of a dose–response effect.

However, it can be noticed that despite twice the number of evoked contractions, the NMES group did not exhibit a doubled total TTI as compared to the NMES + MI group. This can be explained by the fact that the intensity used during NMES to match 20% MVC was set only on one contraction and was not readjusted during the session. Thus, this level of 20% MVC was not maintained during the whole NMES session. The decrease in force can be due on one hand to fatigue caused by the non-physiological (spatially fixed and synchronous) motor unit recruitment (Gregory and Bickel 2005; Bickel et al. 2011) as compared to voluntary contractions (Theurel et al. 2007) and on the other hand to a reduction in the number of motor units recruited due to changes in axonal excitability (Matkowski et al. 2015). Conversely, in the NMES + MI group, sets of 5 NMES contractions were alternated with sets of 5 imagined contractions. This recovery allowed NMES contractions to remain closer to 20% MVC along the session. But still, this muscular treatment was not a sufficient stimulus to induce significant neuromuscular plasticity and strength gains in the combined group as opposed to the NMES-training group. The lack of effect in the combined group could corroborate the dose–effect response of MI training programs (Paravlic et al. 2018), the duration and number of repetitions provided in this group being insufficient to provide the usually observed MI-induced plasticity.

The second hypothesis of this lack of effect in the NMES + MI group lies in a possible antagonist effect of NMES and MI, which possibly canceled the expected summation of NMES and MI effects. As mentioned above, the afferent feedback induced by NMES led to a modulation of a larger loop involving a cortical effect. This supraspinal influence of NMES might also compete with the central activation by MI, since it could be qualitatively different. Indeed, NMES brain activation lies in the relationship between sensory and motor regions, while MI reflects activation directly initiated in the cortex. While several cortical inhibitory mechanisms were proposed during movement preparation of imagined action to prevent the movement from happening (Lebon et al. 2019), this might reveal as contradictory influence between the sensory and motor information provided by NMES. At spinal level, the concurrent effect of MI and NMES is even more apparent, as illustrated by the opposite effects of NMES and MI observed on H-reflex recruitment curves. If MI and NMES might involve the same spinal circuitry, i.e., the presynaptic inhibitory processes, the direction of the modulation is opposite between these two forms of treatment of the spinal cord. While NMES was shown to induce acute increase in spinal presynaptic inhibition (Grosprêtre et al. 2018), in contrast the effect of an acute MI session led to the removal of presynaptic inhibition (Grosprêtre et al. 2019). Therefore, when implemented in the same training session, both modalities might compete, canceling the effect that one should have when performed alone. The acute effect of this combination should be assessed in further research.

Another combination design simultaneously combining MI and NMES, i.e. imagining the contraction while it is electrically evoked, was acutely tested by Kaneko and colleagues (Kaneko et al. 2014). They showed that corticospinal tract excitability was acutely increased when NMES and MI were used simultaneously. MEP amplitude reached a level similar to that measured during brief voluntary muscle contraction, even though voluntary muscle contractions were absent during the combined condition. This could then be an alternative to combine efficiently NMES and MI.

## Practical recommendations

Improving plantar flexors neuromuscular function without involving an important training load is of importance, particularly for frail populations. This muscle group contributes to many functional tasks, more particularly related to balance in upright standing posture. In that matter, plantar flexors MVC appeared to be a critical factor for falling in the elderly (Cattagni et al. 2014).

If reaching functional arousal such as increasing maximal force, the present study raised that MI and NMES can lead to similar gains. Therefore, the choice of one or the other modality could depend upon the strength and weakness of each method. While being very effective to induce neuromuscular plasticity, NMES needs specific material and is subjected to a particular expertise of the operator (electrodes positioning, setting of the device) and more importantly, present the inherent drawback of being uncomfortable for many participants, even when not using a maximal tolerable intensity. On the opposite, MI represents a costless, safe and simple method to induce strength gains. But one of the main drawbacks of MI lies in the expertise of the participant itself to mentally represent an action, and in its commitment to perform the task. Besides being easily boring, which can lead to a decrease in the participant’s dedication to perform the task due to motivational aspects, MI represents a high cognitive process that requires learning and might be hardly manageable for populations with mental disabilities. Therefore, both MI and NMES have their specificities and might be chosen according to the context and the targeted population. But the present study raised that MI and NMES involve, above all, different mechanisms in the training induced plasticity. This should also be considered when choosing to implement one or the other modality for training purpose. While NMES seems to be a very effective stimulus to provide changes at the level of the neuromuscular junction, even in absence of muscle structural changes, MI mostly provides central plasticity.

While combining MI with other sensorial inputs has been widely recommended to magnify the effect of such training modality, the present study raised that antagonist effect might also interfere with the gains in motor performance that should be emphasized by such a modality of training.

## Conclusion and perspectives

This study showed similar gains in MVC between NMES and MI trainings, both relying on neural adaptations which, overall, involved a larger part of the corticospinal system than usually expected, including both supraspinal and spinal plasticity.

Unexpectedly, alternating NMES and MI in the same training session did not imply strength gains, possibly explained by different hypotheses, such as (1) the insufficient stimulus provided at the peripheral level for NMES and at central level for MI or (2) concurrent effects between both modalities.

It is therefore suggested that the present MI and NMES protocols performed alone are more efficient than alternating half of both in a similar training design. Yet, another training design simultaneously combining MI and NMES, i.e. imagining the contraction while it is electrically evoked, might be an alternative. Nonetheless, the acute or chronic effects of repeating this modality of NMES and MI combination have not been investigated and could be the subject of future research.

## Data Availability

The results of the study are presented clearly, honestly, and without fabrication, falsification, or inappropriate data manipulation. All data are available if needed.

## Abbreviations

ANOVA: Analysis of variance
Ca^2^^+^: Calcium ion
CON: Control group
EME: Electromechanical efficiency
EMG: Electromyography
FL: Fascicle length
GL: Gastrocnemius lateralis
GM: Gastrocnemius medialis
*H*_MAX_: Maximal H-reflex
*H*_SUP_: Superimposed H-reflex
*M*_ATH_: M-wave recorded with the corresponding H-reflex
MI: Motor imagery
MIQ-R: Motor Imagery Questionnaire—Revised
*M*_MAX_: Maximal M-wave
*M*_SUP_: Superimposed M-wave
MT: Muscle thickness
NMES: Neuromuscular electrical stimulation
Pα: Pennation angle
Pt: Peak twitch
SOL: Soleus muscle
TA: Tibialis anterior
TTI: Torque time integral
